# BK Polyomavirus Subtypes II and IV in Hematopoietic Cell Transplant Recipients

**DOI:** 10.1128/mra.01053-21

**Published:** 2022-01-06

**Authors:** Elizabeth A. Odegard, Heidi L. Meeds, Steven B. Kleiboeker, Assem Ziady, Anthony Sabulski, Sonata Jodele, Alix E. Seif, Stella M. Davies, Benjamin L. Laskin, Jason T. Blackard

**Affiliations:** a Division of Digestive Diseases, University of Cincinnati College of Medicine, Cincinnati, Ohio, USA; b Eurofins Viracor Laboratories, Lee’s Summit, Missouri, USA; c Department of Pediatrics, University of Cincinnati College of Medicine, Cincinnati, Ohio, USA; d Division of Bone Marrow Transplantation and Immune Deficiency, Cincinnati Children’s Hospital Medical Center, Cincinnati, Ohio, USA; e Perelman School of Medicine, University of Pennsylvania, Philadelphia, Pennsylvania, USA; f Division of Oncology, Children's Hospital of Philadelphia, Philadelphia, Pennsylvania, USA; g Division of Nephrology, Children's Hospital of Philadelphia, Philadelphia, Pennsylvania, USA; DOE Joint Genome Institute

## Abstract

Symptomatic BK polyomavirus (BKPyV) infections are common and relevant in immunocompromised patients. Here, we present full-length BKPyV genomes from samples from patients who received hematopoietic cell transplants in the United States. These individuals had non-subtype I BKPyV, as determined by amplification, next-generation sequencing, and phylogenetic analysis.

## ANNOUNCEMENT

BK polyomavirus (BKPyV), of the family *Polyomaviridae* and the genus *Betapolyomavirus*, is a circular, double-stranded DNA virus that is ubiquitous worldwide and infects up to 90% of the population by 10 years of age (1, 2). In immunosuppressed individuals, primary infection or reactivation of existing virus can cause kidney or bladder injury, which is typically observed in kidney or hematopoietic stem cell transplant recipients. At least four genotypes of BKPyV exist and likely impact viral kinetics, tropism, and pathogenesis. Various studies have identified subtype I as the most prevalent subtype in the United States, although those reports typically include small sample sizes (3). Thus, there is a need to increase the amount of sequencing data for BKPyV to understand more effectively how BKPyV diversity impacts disease. Here, we present two non-subtype I BKPyV strains that were obtained from patient urine samples within the United States.

A BKPyV genome analysis was performed with samples from two subjects from a large cohort study, as reported previously (4). The two patients received hematopoietic cell transplants (HCTs) at Cincinnati Children’s Hospital Medical Center (CCHMC) between April 2013 and May 2018. BKPyV viruria and viremia were quantified in urine samples (limit of detection of 500 copies/ml to 1 × 10^10^ copies/mL) and blood samples (limit of detection of 39 copies/ml to 1 × 10^10^ copies/mL) at Eurofins Viracor Laboratories. The CCHMC institutional review board approved the study, and the patients or their parents/guardians provided written informed consent/assent.

Subject 1 was a 15-year-old Caucasian male, born in the United Arab Emirates (UAE), who received a myeloablative allogeneic bone marrow transplant. Before the transplant, both viruria and viremia were present; at 1 month posttransplant, low-level viremia persisted and viruria was 11,000,000 copies/mL. Subject 2 was a 10-year-old Caucasian male, born in the United States, who received a myeloablative bone marrow transplant. BKPyV viremia and viruria were negative prior to the transplant; at 1 month posttransplant, viremia was 87 copies/mL and viruria was >10^10^ copies/mL.

Urine samples at 1 month posttransplant were processed according to published methods (5). Briefly, the viral DNA was extracted from BKPyV-positive urine samples utilizing the Qiagen QIAamp UltraSens virus kit. One microliter of extracted DNA was used for rolling circle amplification (RCA) with the TempliPhi amplification kit, incubated at 30°C overnight, and linearized with BamHI. A full-length BKPyV PCR was performed using the Qiagen UltraRun LongRange PCR kit and BKPyV-specific primers BK1731F and BK1739R (6). The PCR product was run on a 1% agarose gel and purified with the QIAquick gel extraction kit.

PCR products were subjected to next-generation sequencing (NGS). Library preparation was performed using the New England BioLabs NEBNext Ultra II FS DNA library preparation kit, and the library was sequenced on an Illumina HiSeq 1000 sequencer with the setting SR 1 × 51 bp. The reads generated were run through FastQC for quality control, and no reads were flagged as poor quality. All tools utilized were run with default parameters. Reads for each sample were then mapped to a reference genome (GenBank accession number V01108 [strain Dunlop]) within UGENE version 37.0, generating a consensus sequence (7). Details about sequence raw and assembled reads are presented in Table 1.

**TABLE 1:** Genome characteristics

Subject	Raw reads		Consensus sequence	
	No. of reads	Avg read depth (×)	Length (bp)	GC content (%)
1	1,179,263	11,671	5,153	39.4
2	167,897	1,661	5,153	39.7

Phylogenetic inference was performed with the samples and a set of BKPyV reference genomes using the Bayesian Markov chain Monte Carlo (MCMC) method. Bayesian Evolutionary Analysis by Sampling of Trees (BEAST) version 1.10.4 was run with a chain length of 200,000,000 (8). Results were visualized in Tracer version 1.7.1 to confirm adequate chain convergence. All estimated sample size values were >700, indicating sufficient sampling. A 10% burn-in within Tree Annotator version 1.10.4 was performed, and the maximum clade credibility tree was visualized in FigTree version 1.4.4.

**FIG 1:**
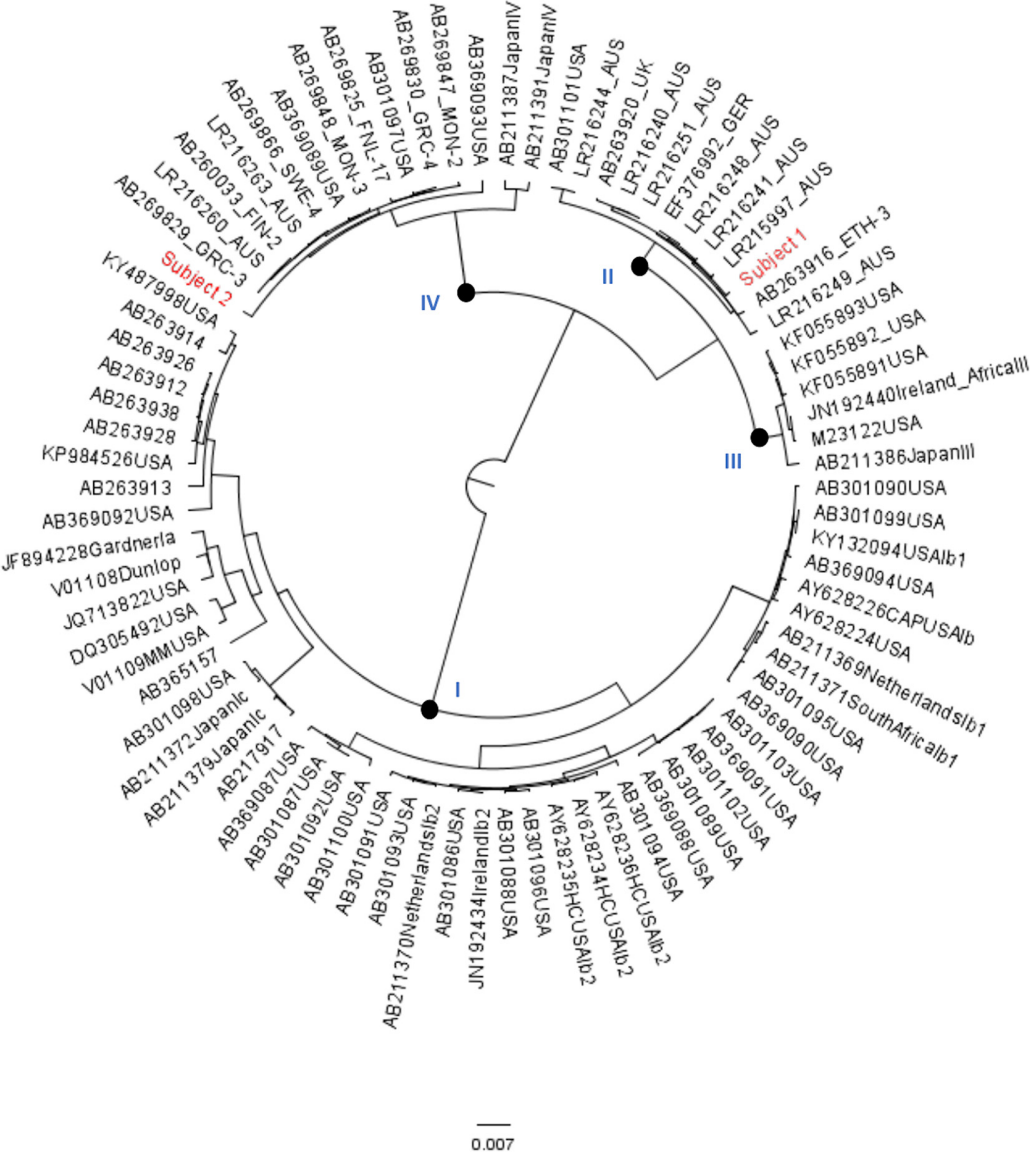
Representative BKPyV full genome sequences (downloaded from GenBank) were aligned, and phylogenetic inference was performed in BEAST version 1.10.4 (8). Sequences are labeled with their GenBank accession number, country of origin, and subtype, if known. Nodes separating subtypes are marked with closed circles and labeled. Sequences of interest (subject 1 and subject 2) are highlighted in red.

Phylogenetic analysis showed that the full-length BKPyV sequence from subject 1 clustered with BKPyV subtype II, while the viral sequence from subject 2 clustered with BKPyV subtype IV (Fig. 1). These subtypes have been noted (but only rarely) within the United States. Thus, there is a need for sequencing of BKPyV strains from large, cohort-based studies to establish a true distribution of subtypes, while considering the birthplace of individuals regardless of the sample collection location. Additionally, increasing the published sequences and sequencing data allows the functional consequences of viral diversity to be investigated more thoroughly. To date, BKPyV full-length genome sequences are limited in number, particularly for non-subtype I subtypes, given that ∼90% of the population has been exposed to BKPyV.

### Data availability.

The raw sequence data are available under BioProject PRJNA670723, with SRA accession numbers SRX11657520 for subject 1 and SRX11657521 for subject 2. The consensus BKPyV genome is available in GenBank under the accession numbers MZ822365 for subject 1 and MZ822366 for subject 2.
